# The role of zinc, albumin, c reactive protein, and interleukin-6 in differentiation of unipolar depression and depression in bipolar disorder

**DOI:** 10.1192/j.eurpsy.2021.917

**Published:** 2021-08-13

**Authors:** T. Bagaric, M. Zivkovic, P. Marinovic, A. Kozmar, N. Jaksic, M. Sagud, A. Mihaljevic-Peles

**Affiliations:** 1 Department Of Psychiatry, University Hospital Center Zagreb, Zagreb, Croatia; 2 Department Of Laboratoy Diagnostics, University Hospital Center Zagreb, Zagreb, Croatia; 3 School Of Medicine, University of Zagreb, Zagreb, Croatia

**Keywords:** depression, bipolar disorder, C-reactive protein

## Abstract

**Introduction:**

There is no clinical difference between depressive episodes in bipolar disorder compared to major depressive disorder, which is why bipolar disorder remains unrecognized. Correctly distinguishing these disorders is of great importance because the therapeutic approach differs significantly. According to previous research, zinc, albumin, C reactive protein (CRP), and interleukin-6(IL-6) seem to play a role in differentiating these two types of depressive episodes.

**Objectives:**

To determine zinc, albumin, CRP and IL-6 serum concentrations in patients with major depressive disorder and depressive episode of bipolar disorder.

**Methods:**

Research involved 60 participants. Participants signed informed consent prior to inclusion in the study. Sociodemographic data have been collected using a previously structured questionnaire. The severity of depressive symptoms has been measured by the Montgomery Asberg Depression Rating Scale (MADRS) and the Hamilton Depression Scale (HAM-D-17). Blood samples were obtained from each study participant’s brachial vein, to determine zinc, albumin, C reactive protein and interleukin-6 serum concentrations.

**Results:**

Statistically significant difference was found in zinc serum levels between the two analysed groups. In the overall sample, there is a significant positive correlation between the results on the rating scales and the serum level of CRP.

**Conclusions:**

We confirmed an association between serum levels of CRP and the severity of the illness. Regardless, these are preliminary results of the research. Sufficient final conclusion cannot yet be drawn because it is being limited by the sample size and further investigation is needed.

